# A consensus list of ultrasound competencies for graduating emergency medicine residents

**DOI:** 10.1002/aet2.10817

**Published:** 2022-11-21

**Authors:** David A. Haidar, William J. Peterson, Patrick G. Minges, Jennifer Carnell, Jason T. Nomura, John Bailitz, Jeremy S. Boyd, Megan M. Leo, E. Liang Liu, Youyou Duanmu, Josie Acuña, Ross Kessler, Marco F. Elegante, Mathew Nelson, Rachel B. Liu, Resa E. Lewiss, Arun Nagdev, Rob D. Huang

**Affiliations:** ^1^ Department of Emergency Medicine Michigan Medicine Ann Arbor Michigan USA; ^2^ Department of Emergency Medicine University of Cincinnati College of Medicine Cincinnati Ohio USA; ^3^ Department of Emergency Medicine Baylor College of Medicine Houston Texas USA; ^4^ Department of Emergency Medicine, Sidney Kimmel Medical College Thomas Jefferson University Philadelphia Pennsylvania USA; ^5^ Department of Emergency Medicine, Feinberg School of Medicine Northwestern University Chicago Illinois USA; ^6^ Department of Emergency Medicine Vanderbilt University Medical Center Nashville Tennessee USA; ^7^ Department of Emergency Medicine Boston University School of Medicine Boston Massachusetts USA; ^8^ Department of Emergency Medicine Emory University School of Medicine Atlanta Georgia USA; ^9^ Department of Emergency Medicine Stanford University School of Medicine Palo Alto California USA; ^10^ Department of Emergency Medicine University of Arizona Tucson Arizona USA; ^11^ Department of Emergency Medicine University of Washington Seattle Washington USA; ^12^ Department of Emergency Medicine University of Florida College of Medicine Gainesville Florida USA; ^13^ Department of Emergency Medicine Zucker Northwell School of Medicine, Northwell Health Manhasset New York USA; ^14^ Department of Emergency Medicine Yale School of Medicine New Haven Connecticut USA; ^15^ Department of Emergency Medicine Highland Hospital, Alameda Health System Oakland California USA; ^16^ Department of Emergency Medicine University of Michigan Medical School Ann Arbor Michigan USA

**Keywords:** consensus, education, graduate medical education, point‐of‐care ultrasound, resident, ultrasound

## Abstract

**Objectives:**

Emergency ultrasound (EUS) is a critical component of emergency medicine (EM) resident education. Currently, there is no consensus list of competencies for EUS training, and graduating residents have varying levels of skill and comfort. The objective of this study was to define a widely accepted comprehensive list of EUS competencies for graduating EM residents through a modified Delphi method.

**Methods:**

We developed a list of EUS applications through a comprehensive literature search, the American College of Emergency Physicians list of core EUS benchmarks, and the Council of Emergency Medicine Residency‐Academy of Emergency Ultrasound consensus document. We assembled a multi‐institutional expert panel including 15 faculty members from diverse practice environments and geographical regions. The panel voted on the list of competencies through two rounds of a modified Delphi process using a modified Likert scale (1 = not at all important, 5 = very important) to determine levels of agreement for each application—with revisions occurring between the two rounds. High agreement for consensus was set at >80%.

**Results:**

Fifteen of 15 panelists completed the first‐round survey (100%) that included 359 topics related to EUS. After the first round, 195 applications achieved high agreement, four applications achieved medium agreement, and 164 applications achieved low agreement. After the discussion, we removed three questions and added 13 questions. Fifteen of 15 panelists completed the second round of the survey (100%) with 209 of the 369 applications achieving consensus.

**Conclusion:**

Our final list represents expert opinion on EUS competencies for graduating EM residents. We hope to use this consensus list to implement a more consistent EUS curriculum for graduating EM residents and to standardize EUS training across EM residency programs.

## INTRODUCTION

Emergency ultrasound (EUS) is a critical component of emergency medicine (EM) resident education.^1^, ^2^ Residents find EUS to be relevant to their future practice and therefore an important skill to develop in training.^3^ Eighty‐eight percent of residency programs in the country have a dedicated EUS rotation.^4^ Given its importance, multiple governing bodies have developed guidelines over the years to help structure how ultrasound training is implemented in graduate medical education (GME).^1^, ^2^ However, there is currently no unified consensus list of competencies for EUS training of residents. As EUS education and training is standardized at the fellow level through the development of the Emergency Ultrasound Fellowship Accreditation Council and the focused practice designation (FPD), it is equally important to develop a standardized process for training residents in EUS.

Multiple prior studies have evaluated methods to implement EUS education in residency programs and to assess competency.^5^, ^6^, ^7^ In 2008, the American College of Emergency Physicians (ACEP) developed EUS policy statements to support the use of point‐of‐care ultrasound as a routine part of EM practice.^1^ This was followed by the development of the Council of Emergency Medicine Residency‐Academy of Emergency Ultrasound (CORD‐AEUS) consensus guidelines for assessment and progression of EUS in 2012, coinciding with the inclusion of EUS in the Accredited Council for Graduate Medical Education (ACGME) milestones.^1^, ^8^ The milestones introduced minimum requirements for EUS completion, but equated the number of ultrasounds performed with competency. While the milestones recommended 150 minimum total focused EUS scans, Blehar et al.^7^ determined that different types of scans required different numbers of minimums for residents to reach proficiency, ranging from 30 to 80 scans per type. Furthermore, as more programs began to implement EUS education into their curricula, the lack of standardization allowed for vast differences in the quality of training. Akhtar et al.^9^ discussed the importance of implementing dedicated EUS training sessions so that residents can utilize EUS in their daily clinical practice, emphasizing that unsupervised image acquisition and interpretation alone is insufficient. Amini et al.^10^ found that there is significant variation in the methods of competency assessment.

Despite the clear importance of EUS in resident education and the vast amount of literature discussing its implementation, there does not appear to be any curriculum standardization or consensus competencies. In 2021, The Milestones 2.0 Project replaced the original ACGME milestones—which eliminated the specific procedural competencies.^11^, ^12^ EUS, along with five other procedural milestones, were combined into one “general approach to procedures.”^11^, ^12^ This change gave programs autonomy in defining basic versus advanced procedures for their given context.^12^ However, there is still no unifying consensus list of competencies for EUS training. Given this, graduating residents will have various degrees of exposure to EUS which could potentially lead to varying levels of skill and comfort.

The objective of this study was to define a widely accepted comprehensive list of EUS competencies for all graduating EM residents through a modified Delphi consisting of a diverse group of leaders in the ultrasound education community. We define competencies in the context of the competency‐based medical education (CBME) framework of Van Melle et al.^13^ as “knowledge, attitudes, or observable behaviors which together account for the ability to deliver a specified professional service,” which originates from the landmark competency‐based curriculum development work of McGahie et al.^14^

## METHODS

### Study design

**FIGURE 1:**
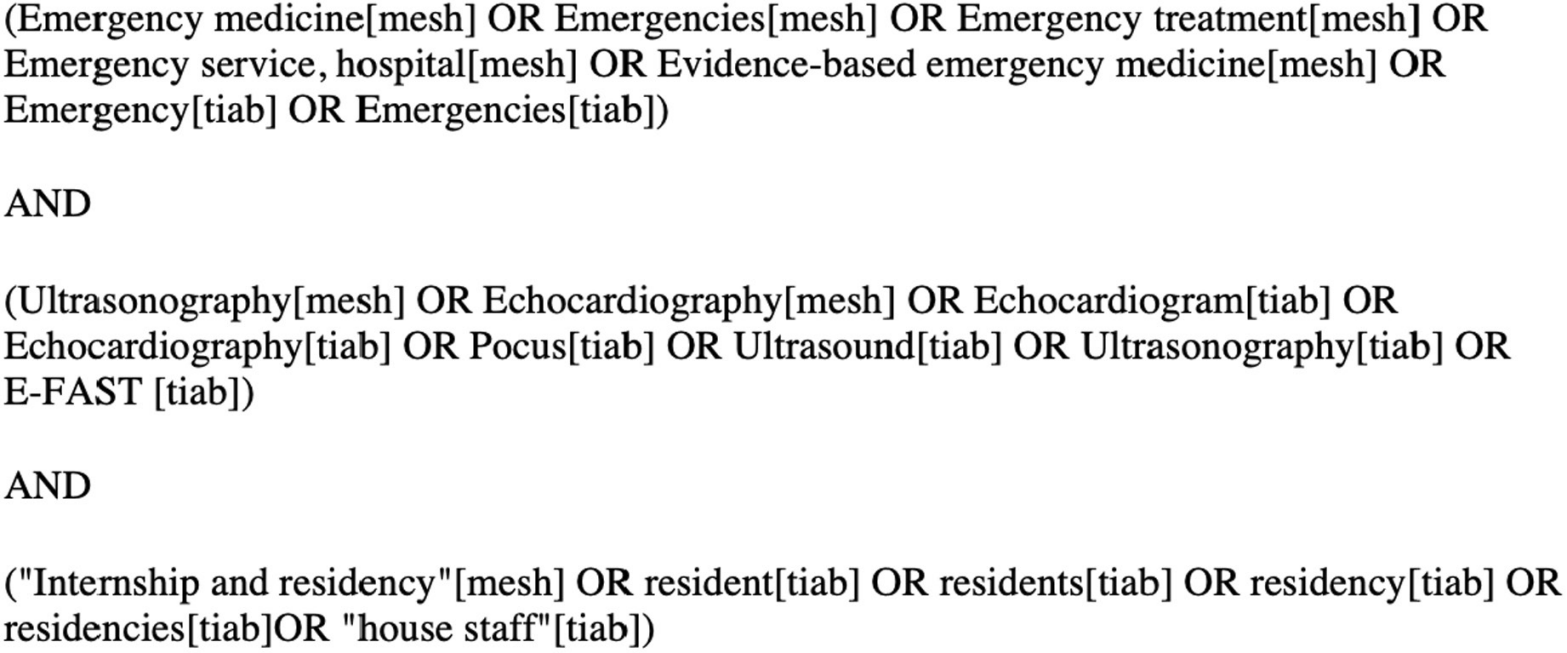
Literature search for comprehensive list of EUS competencies

We developed an extensive list of relevant topics within EUS using the ACEP list of ultrasound guidelines and CORD‐AEUS consensus recommendations and input from a group of educationally focused ultrasound faculty (including one program director, EUS fellowship director, and EUS director) and EUS fellows at a large academic EM residency in the central region of the United States.^3^, ^8^ After this, we conducted a comprehensive literature review of all clinical applications of ultrasound with the assistance of a librarian to develop a final comprehensive list of applications in Fall 2020.^15^, ^16^, ^17^, ^18^, ^19^, ^20^, ^21^, ^22^, ^23^, ^24^, ^25^, ^26^, ^27^, ^28^, ^29^, ^30^, ^31^, ^32^, ^33^, ^34^, ^35^, ^36^, ^37^, ^38^, ^39^, ^40^, ^41^, ^42^, ^43^, ^44^, ^45^, ^46^, ^47^, ^48^, ^49^, ^50^, ^51^, ^52^, ^53^, ^54^, ^55^ The specific search terms are presented in Figure 1. The final list included topics such as physics and general principles as well as normal anatomy and pathology in the following categories: trauma, aorta, thoracic, cardiac, obstetrics and gynecology (OBGYN), testicular, ocular, neurology, venous, biliary, renal, soft tissue and musculoskeletal (MSK), head and neck, bowel, procedural guidance, and airway. This study was ruled exempt and not regulated by our institutional review board (HUM00197359).

### Study setting and participants

We assembled a multi‐institutional expert panel of 15 faculty members from 15 programs across the country. We targeted ABEM‐certified and fellowship‐trained ultrasound experts with extensive experience in EUS education at the GME level from diverse practice environments and with varying years of experience. Panelists self‐described their practice type as community, county, academic, or a combination. Six programs had multiple designations. Our panel consisted of two former program directors, two former assistant program directors, seven current or former ultrasound directors, and nine current or former EUS fellowship directors. We used the Association of American Medical Colleges Residency Explorer tool to define each program's geographic region. Panelist demographics are described in Table 1. We used a modified Delphi technique, which was decided a priori, to rate each item in the list of EUS applications. The modified Delphi is a well‐established and theory‐driven method with validity evidence used to achieve expert opinion.^56^, ^57^ We created an online questionnaire of EUS applications using the Qualtrics online platform, which allowed us to send, receive, and track information from individual participants and confidentially store data. The questionnaire also allowed for experts to provide commentary regarding question clarification or general thoughts.

**TABLE 1:** Panelist demographics

Sex	
Female	7
Male	8
Years of experience, mean (range)	10.63 (2–20)
Practice type	
Academic	9
Community hybrid	1
County hybrid	5
Geographic region	
Northeast	5
Central	2
Southern	4
Western	4

### Measurements and outcomes

During the first round, we asked panelists to rate how important each EUS application is for “EM residents to be able to competently perform at the time of graduation.” We utilized a 5‐point Likert scale to quantify this (1 = not very important, 2 = not important, 3 = kind of important, 4 = important, 5 = very important).

After the first round, we extracted the data from Qualtrics and generated detailed reports that we sent to the individual panelists. These reports included data for each individual EUS application such as individual panelist's response, the group mean, standard deviation (SD), and level of agreement. Levels of agreement as outlined by de Loe^58^ were calculated and used to assess modified Delphi results and achieve consensus, which is a validated approach to interpret modified Delphi data.^59^ Levels of agreement are broken down into high, medium, and low agreement. High agreement was defined as when >80% of responses fell on two continuous points on the 5‐point Likert scale. Medium agreement was defined as between 70% and 79.99%. Low agreement was defined as below 70%. Based on these guidelines, items qualified for inclusion in the consensus guidelines when >80% of responses were either a 4 or a 5.

After panelists received the results of the first round, we conducted two separate virtual meetings to ensure maximum participation from panelists. We also had a running online document available for meeting minutes so that those unable to attend the virtual meetings could still participate by providing commentary. Nine of 15 panelists and the three nonpanelist authors participated in the virtual meetings. The remaining six panelists were able to provide commentary on the online document asynchronously. During these meetings, panelists discussed the results of the first round of voting. They provided general opinions on the consensus list, discussed the results, and suggested changes to the survey. The discussions specifically focused on medium agreement topics, low agreement topics with high means, and high agreement topics with low means.

### Data analysis

After these meetings, we revised the questionnaire to include changes from the discussion and included the group mean with each individual application. This second questionnaire was sent to panelists again using the Qualtrics online platform. After panelists completed the second questionnaire, the final results were analyzed using the above methods to assess mean, SD, and level of agreement. The applications that achieved high agreement after the second round constituted the final consensus list of applications. Medium agreement and low agreement items were eliminated from the final list.

## RESULTS

The first questionnaire consisted of 359 applications of EUS. Fifteen of 15 (100%) panelists completed this initial questionnaire. A total of 195 applications achieved high agreement, four achieved medium agreement, and 160 achieved low agreement. The results are available in Data Supplement 1.

After the virtual meetings, we removed three questions and added 13 questions. We removed three questions from the pulmonary pathology section as they focused on specific medical diagnoses and replaced them with questions that focused on specific sonographic findings instead. We added questions to the following categories: pulmonary pathology, cardiac pathology, OBGYN normal anatomy, venous pathology, renal pathology, soft tissue pathology, intraoral pathology, and procedural guidance. New questions were added to broaden our scope to include additional pathologies or EUS applications.

The second questionnaire consisted of 369 applications of EUS and 100% of the panelists (15/15) completed this as well. A total of 209 applications achieved high agreement, nine achieved medium agreement, and 151 achieved low agreement. The results of the second round are available in Data Supplement 1. Our expert panel included a total of 209 EUS applications in the final consensus list of EUS applications for graduating EM residents, which are available in Table 2.

**TABLE 2:** Final consensus list of EUS competencies

General principles
Identifies need to accurately label images with patient information
Places patient in appropriate position
Explains the exam to be performed to the patient
Selects appropriate probe
Selects appropriate exam preset
Adjusts depth to adequately identify all relevant anatomy and pathology
Adjusts gain to appropriately identify all relevant structures and pathology
Assures proper cleaning of probe before and after use
Relays findings to patient care team
Appropriately documents all findings in the medical record
Physics
Identifies posterior acoustic enhancement
Identifies acoustic shadowing
Identifies mirror artifact
Trauma: normal anatomy
Identify liver
Identify kidney
Identify diaphragm
Identify spine
Identify Morison's pouch
Identify splenorenal space
Identify splenodiaphragmatic space
Identify paracolic gutter bilaterally
Identify pleural space bilaterally
Identify bladder in transverse and sagittal planes
Identify uterus in a female patient
Identify prostate in a male patient
Identify pouch of Douglas in a female patient
Identify rectovesicular space in a male patient
Identify ribs
Identify lung pleura
Identify lung sliding
Trauma: pathology
Intraabdominal hemorrhage
Identify the anechoic appearance of intraabdominal free fluid
Identify the hypoechoic/mixed echogenic appearance of clotting intraperitoneal blood
Identify potential spaces where intraabdominal blood can accumulate
Pneumothorax
Identify loss of lung sliding
Identify the appearance of the lung point
Pleural effusion/hemothorax
Identify the pleural space where pleural fluid will accumulate
Identify the appearance of anechoic pleural fluid
Identify the appearance of mixed‐echogenicity complex pleural fluid
Identify the spine sign
Aorta: normal anatomy
Identify aorta in transverse, sagittal, and coronal plane
Identify spine
Identify IVC
Identify celiac axis
Identify SMA
Identify aortic bifurcation
Aorta: pathology
Aortic aneurysm
Measure abdominal aorta in transverse in proximal, mid, and distal abdomen
Measure outer to outer wall in anterior to posterior plane
Measure outer to outer wall
Aortic dissection
Identify aortic dissection flap
Measure aortic root
Lung: normal anatomy
Identify the different zones of the lung (upper/mid/lower)
Identify A‐lines
Lung: pathology
Interstitial pulmonary fluid
Identify B‐lines
Identify differential for diffuse bilateral B‐line pattern
Identify differential for focal bilateral B‐line pattern
Pneumonia
Identify pneumonia pattern of focal B‐line appearance
Identify lung consolidation
Identify subpleural effusion
Cardiac: normal anatomy
Identify RA in apical four chamber and subxiphoid views
Identify RV in parasternal long, parasternal short, apical four chamber, and subxiphoid views
Identify LA in parasternal long, apical four chamber, and subxiphoid views
Identify LV in parasternal long, parasternal short, apical four chamber, and subxiphoid views
Identify aortic outflow tract in parasternal long and apical four chamber views
Identify descending thoracic aorta in parasternal long view
Identify mitral valve in parasternal long, parasternal short, apical four chamber, and subxiphoid views
Identify tricuspid valve in apical four chamber and subxiphoid views
Identify aortic valve in parasternal long, apical four chamber, and subxiphoid views
Identify papillary muscles
Identify pericardium
Identify IVC in long and short axis
Identify hepatic vein confluence with IVC
Cardiac: pathology
Cardiac arrest
Identify sonographic appearance of cardiac standstill
Identify sonographic appearance of ventricular fibrillation
Identify sonographic appearance of agonal cardiac activity
Decreased ejection fraction
Accurately characterize ejection fraction into hyperdynamic/normal/mildly depressed/moderately depressed/severely depressed using subjective interpretation of cardiac contraction
Cardiac tamponade
Identifies where pericardial fluid accumulates
Identifies right atrial collapse
Identifies right ventricular collapse
Identifies plethoric IVC
Valvular
Identifies vegetation on valve
Measures aortic root diameter
Volume assessment
Demonstrate evaluation of IVC collapsibility 2 cm inferior from the confluence of the hepatic veins
Identifies plethoric IVC
Identifies collapsed IVC
Elevated right heart pressure
Identifies the D sign
Identifies an increased RV:LV ratio
Myocardial infarction
Identifies the septal wall of the heart on parasternal short
Identifies the anterior wall of the heart on parasternal short
Identifies the lateral wall of the heart on parasternal short
Identifies the posterior wall of the heart on parasternal short
Identifies the inferior wall of the heart on parasternal short
OBGYN: normal anatomy
Identifies uterus in transverse axis using transabdominal approach
Identifies uterus in the sagittal axis using the transabdominal approach
Identifies uterus in the coronal axis using the transvaginal approach
Identifies uterus in the sagittal axis using the transvaginal approach
Identifies bladder
Identifies ovaries
Identifies right ovary
Identifies left ovary
Identifies pouch of Douglas
Identifies endometrial stripe
Identifies cervix
First‐trimester intrauterine pregnancy
Identifies gestational sac
Identifies yolk sac
Identifies fetal pole
Identifies fetal heart rate
Measure fetal heart rate using M‐mode
Performs crown–rump length measurement to estimate gestational age
OBGYN: pathology
Ovarian cyst
Identifies echogenic fluid in the cul‐de‐sac
Ectopic pregnancy
Identifies empty uterus in setting of positive pregnancy test
Identifies pseudo‐gestational sac in setting of positive pregnancy test
Identifies yolk sac/fetal pole in a nonuterine location
Identifies free fluid in the pouch of Douglas
Identifies free fluid in Morison's pouch
Testicular: normal anatomy
Identifies testicle
Ocular: normal anatomy
Identifies anterior chamber
Identifies posterior chamber
Identifies pupil
Identifies lens
Identifies optic nerve sheath
Ocular: pathology
Posterior chamber
Identifies vitreous hemorrhage
Identifies vitreous detachment
Identifies retinal detachment
Distinguishes vitreous from retinal detachment via visualization of optic nerve sheath
Demonstrates dynamic evaluation of the eye via patient eye movement
Other
Identifies globe rupture
Venous: normal anatomy
Identifies greater saphenous vein
Identifies common femoral vein
Identifies deep femoral vein
Identifies superficial femoral vein
Identifies popliteal vein
Identifies popliteal trifurcation (anterior tibial, posterior tibial, peroneal)
Venous: pathology
DVT
Identifies compressibility of a normal vessel
Identifies lack of compressibility in the setting of a DVT
Performs compression at areas of venous bifurcation
Identifies presence of DVT in lower extremity
Biliary: normal anatomy
Identifies liver
Identifies gallbladder in long axis
Identifies gallbladder in short axis
Identifies portal triad
Identifies portal vein
Identifies hepatic artery
Identifies common bile duct
Biliary: pathology
Gallstones
Identifies gallstones
Identifies gallstone characteristics of echogenicity, shadowing, and mobility
Identifies WES sign (wall–echo–shadow)
Identifies gallbladder sludge
Cholecystitis
Identifies pericholecystic fluid
Identifies increase size of gallbladder wall
Measures anterior gallbladder wall in short axis
Identifies sonographic Murphy's sign
Choledocholithiasis/cholangitis
Identifies enlarged common bile duct
Measures common bile duct
Renal: normal anatomy
Identifies renal cortex
Identifies renal medulla
Identifies renal pelvis
Identifies ureter
Identifies bladder
Renal: pathology
Identifies mild hydronephrosis
Identifies moderate hydronephrosis
Identifies severe hydronephrosis
Identifies mimics of hydronephrosis
Uses color to differentiate hydronephrosis from vasculature
MSK: normal anatomy
Identifies muscle appearance
Identifies tendon appearance
Identifies peripheral nerve appearance ‐ anisotropy
Identifies peripheral nerve apperance ‐ hyperechoic honeycomb
Identifies normal appearance of dermis
Identifies appearance of lymph node
Identifies bone as hyperechoic line in longitudinal and transverse planes
MSK: pathology
General
Identifies joint dislocation
Identifies joint effusion
Soft tissue
Identifies cobblestoning
Identifies other causes of cobblestoning—lymphedema/edema
Identifies appearance of abscess
Identifies air echoes as sign of necrotizing fasciitis
Identifies foreign objects in subcutaneous tissue
Shoulder
Identifies humeral head
Identifies glenoid
Knee
Identifies patella
Identifies femur
Identifies tibia
Identifies patellar tendon
Ankle
Identifies Achilles tendon
Identifies Achilles tendon rupture
Head and neck
Identifies appearance of peritonsillar abscess using endocavitary probe
Bowel: pathology
SBO
Identifies to and fro peristalsis as sign of SBO
Procedures
Needle guidance
Identifies and tracks needle in long axis
Identifies and tracks needle tip in short axis
Identifies important nearby structures
Confirms location of guidewire within vessel
Confirms location of catheter within vessel
Correctly identifies appropriate vessel
Correctly identifies size and location of vessel
Identifies back‐walling of needle or guidewire in vessel
Identifies needle going through and through vessel
Fluid drainage
Identifies anechoic or hypoechoic fluid pocket
Procedures
Can place internal jugular CVC under ultrasound guidance
Can place femoral vein CVC under ultrasound guidance
Can place radial arterial line under ultrasound guidance
Can cannulate vessel in short and long axis
Can place femoral arterial line under ultrasound guidance
Can perform thoracentesis under ultrasound guidance
Can perform paracentesis under ultrasound guidance
Can perform paracentesis with ultrasound assistance
Can perform arthrocentesis under ultrasound guidance
Can perform pericardiocentesis under ultrasound guidance
Can perform nerve blocks under ultrasound guidance
Can place peripheral venous line under ultrasound guidance

Abbreviations: CVC, central venous catheter; DVT, deep venous thrombosis; IVC, inferior vena cava; LA, left atrium; LV, left ventricle; MSK, musculoskeletal; OBGYN, obstetrics and gynecology; RA, right atrium; RV, right ventricle; SBO, small bowel obstruction; SMA, superior mesenteric artery.

The topics included in the final list are general principles, physics, normal trauma anatomy and trauma pathology, normal aorta and aorta pathology, normal lung and lung pathology, normal cardiac and cardiac pathology, normal OBGYN and OBGYN pathology, normal testicular anatomy, normal ocular and ocular pathology, normal venous anatomy and venous pathology, normal biliary and biliary pathology, normal renal and renal pathology, normal MSK and MSK pathology, head and neck pathology, bowel pathology, and procedures. Organ systems with no elements in the final list were neurology, airway ultrasound, normal bowel anatomy, and testicular pathology.

## DISCUSSION

Our comprehensive list includes elements of all the core EUS applications from both the ACEP EUS guidelines and the CORD‐AEUS consensus document.^1^, ^8^ Adjunct applications from the ACEP EUS guidelines that overlapped included advanced echo, small bowel obstruction (SBO), adnexal pathology, and testicular pathology.^1^ Our list expanded on these core topics to include specific details outlining normal anatomy and pathology, with the aim of providing a more comprehensive list that better informs institutions on what topics to include in an EUS curriculum. It is not surprising that our list includes more EUS topics than the CORD‐AEUS consensus document from 2011.^8^ While both projects aimed to define expectations for graduating EM residents, the footprint of ultrasound within EM has changed dramatically over the past decade. Every year, the EM workforce includes a higher percentage of working emergency physicians for whom EUS was a required part of their EM residency curriculum. Additionally, the field of EUS itself has changed—a decade ago EUS was relatively new to EM when compared to topics long included in the EM scope of practice. Today, EM physicians have a pathway to an ABEM FPD in advanced emergency ultrasonography. The increasing number of topics our study generated may be due to a natural maturation and expansion of EUS. As with many aspects of medicine, what was once cutting edge has become routine.

Conversely, it is not surprising that some elements included within the ACEP EUS guidelines and the CORD‐AEUS consensus document failed to make our consensus list, as the aim of our study is fundamentally different. The ACEP guidelines describe the potential scope of ultrasound use within the clinical practice of EM. Our goal was to define a minimum expected EUS competency for EM residents graduating from an ACGME‐accredited training program at time of graduation. Our exclusion of bowel ultrasound (other than SBO), transesophageal echo, contrast‐enhanced ultrasound, and transcranial Doppler is not a break from the ACEP guidelines, but rather a recognition that competency in these specific EUS applications may require additional focus and training within or after residency. Furthermore, the exclusion of these applications and other advanced skills from the CORD‐AEUS consensus document is consistent with the goal of our project to define a list of EUS competencies for all graduating EM residents—as competency in such advanced topics is likely to require participation in advanced tracks or additional training opportunities that may not be available to all EM residents.

Our panel did not include pediatric EM–trained physicians, which may explain why bowel pathologies outside of SBO did not meet consensus criteria for inclusion—as the use of ultrasound to diagnose intussusception, appendicitis, and pyloric stenosis is more prevalent in pediatric populations. This may also reflect the challenge of performing sufficient pediatric ultrasound studies to develop competency, which is a challenge faced even by pediatric EM fellows who spend their clinical time in the pediatric environment.^60^

The organ systems included in our consensus list represent the most common types of exams completed in the emergency department.^61^ A recurring theme during our discussion was the consistent expectation that residents should be able to recognize the absence of normal anatomy and function as opposed to specific pathologic diagnoses. Residents are expected to recognize basic anatomy so that they do not overcall normal variants or normal findings as pathology. Furthermore, the general consensus was that residents should not be expected to specifically identify all of these abnormalities but should instead recognize that an abnormality exists and appropriately follow up with further imaging, consultation, or additional workup. While our procedures section specifically listed individual procedures, we did not specifically mention individual regional nerve blocks and instead chose to include an all‐encompassing question because previous studies have already defined an ultrasound guided regional nerve block curriculum using a modified Delphi technique.^62^

Our Delphi group had a robust discussion about the physics topics included in our questionnaire. Unsurprisingly, ultrasound experts considered understanding of common artifacts to be an important and clinically relevant skill for EM residents to avoid misdiagnosing artifacts as pathology and to also recognize normal anatomy accurately. There was significant discussion on whether the recognition of artifact alone was sufficient, compared to the true understanding of the physics behind it, with the group's opinion being split evenly on the matter. Side lobe artifact and aliasing both did not reach consensus, despite the panel agreeing they were important topics to be familiar with.

There was a lengthy discussion regarding the importance of teaching transvaginal ultrasound (TVUS) at the resident level. While the questionnaire did not specify image acquisition and interpretation via transabdominal or transvaginal approach, the panel agreed that it was important for residents to be able to recognize images obtained via TVUS, but not necessarily expected that residents would perform high volumes of TVUS during their residency. This led to the important conversation of utilizing simulation for rare sonographic findings or pathologies when clinical practice was not sufficient in providing these experiences. Where programs cannot support a robust simulation curriculum, they can instead utilize structured online courses and the vast number of free open‐access medical education resources such as podcasts, blog posts, instructional videos, etc. Another interesting finding was that residents were expected to identify the presence of lower extremity deep venous thrombosis (DVTs) but not upper‐extremity DVTs, potentially due to the complexity in diagnosing upper extremity DVTs, the controversial management, and the fact that they are less prevalent.

Topics that were excluded from the final list include testicular pathology, normal bowel anatomy, neurology, and airway ultrasound. While normal testicular anatomy was included, testicular pathology did not reach consensus. Our questions specifically asked if residents could identify hydroceles, varicoceles, epididymitis, orchitis, or hernias. If we instead phrased the questions as recognizing presence of fluid or inflammation, this may have led to higher agreement and possible inclusion into the consensus list. This is likely related to the recurring theme that recognition of absence of normal is more important than diagnosing specific abnormalities. In line with this theme, we likely did not include any normal bowel anatomy given that it is more important to recognize presence or absence of dilated loops of bowel than it is to be able to identify specific anatomic structures of normal bowel.

The neurology section specifically focused on spinal anatomy and lumbar punctures, and the consensus was that use of ultrasound for lumbar puncture was not essential. Finally, airway anatomy was not included, and discussion among panelists was that ultrasound use in airway management was not a resident‐level expectation but rather a fellow‐level skill. There was commentary that recognizing airway structures was important so that one recognizes what structures to avoid during needle insertion for central line access. Comments from the Round 2 survey mainly focused on question phrasing and rewording certain topics, but there were no significant additions or changes included.

Our consensus list serves as an initial benchmark for graduating residents. Future studies could explore both in theory and in practice how these competencies fit into the broader CBME framework.^13^ The CBME framework includes five core components: outcome competencies, progressive sequencing of competencies, tailored learning experiences, competency‐focused teaching instruction, and programmatic assessment.^13^ This study provides a list of outcome competencies satisfying the first of the five core components. Further work could be done to sequence these, develop and implement learning experiences for learners to achieve these competencies, develop teaching practices to promote the development of these competencies, and to develop a programmatic assessment piece to support and document the developmental acquisition of these competencies in resident learners. Future studies could also explore additional outcomes after implementation of a curriculum to teach to these competencies—such as changes in number of scans completed, scans billed, and number of confirmatory studies ordered after benchmark implementation. Patient‐centered outcomes such as changes in management could also be considered.

## LIMITATIONS

Despite our comprehensive literature search with the help of a librarian, our consensus list may not have been exhaustive. By allowing panelists to provide suggestions during the survey and during our discussion, we attempted to maximize the number of topics included. Additionally, attempting to include an exhaustive and large list of items for our expert panel to address in each round may have contributed to survey fatigue and decreased attention to detail in responses compared to a smaller list of items.

There are inherent limitations to using Delphi panels due to the potential for bias. We attempted to mitigate this by including a diverse group of panelists from various geographic locations, practice environments, years of experience, and institutional roles. However, our panel of experts may not have been representative of all residency programs throughout the country. We did have representation from a mix of community, county, and academic programs. However, our panel predominantly came from academic programs, which may not fully represent the opinion on training at exclusively community or county sites. Given this, certain EUS applications that did not reach consensus may be more important to programs where ultrasound techs or consultants are not as readily available. Conversely, if our expert panel does not adequately represent the community or county consensus, there may be some items included in the final list that certain programs may not find as useful. Furthermore, our panel consisted mostly of programs that support an EUS fellowship, and since residents complete more scans when an EUS fellowship is present at their program, this introduces another bias toward the breadth of competencies selected.^63^

While some of our ultrasound faculty are involved in program leadership, we did not include non–ultrasound‐trained program directors, department chairs, residents, or others who may have interest in resident ultrasound training as they were less likely to have predictable knowledge or experience developing an EUS curriculum for residents. Pediatric emergency medicine (PEM) faculty representation was also not included in this process. Future work could examine EUS items pertinent to PEM at the resident or fellow level.

While this study focused on developing a consensus list of EUS competencies, this consensus list requires further validation and feasibility testing. This can be accomplished by developing curricula using this list and obtaining learner and faculty feedback. There may be barriers to implementing and evaluating a curriculum based on this extensive set of competencies depending on resources available at an individual residency program. Potential barriers include limitations in dedicated time for EUS education, adequate number of machines to scan on shift, faculty with EUS training and comfort teaching the above topics, and the ability to supplement clinical learning with simulation for more rare pathologies or clinical presentations.

## CONCLUSIONS

In summary, our final consensus list represents expert opinion on emergency ultrasound competencies for graduating emergency medicine residents. We hope to use this consensus list as a guide for programs to develop a more consistent and robust emergency ultrasound curriculum for future graduating emergency medicine residents and to standardize residency emergency ultrasound training across a diverse group of emergency medicine training programs.

## AUTHOR CONTRIBUTIONS

David A. Haidar, William J. Peterson, and Rob D. Huang proposed goals and designed the study. William J. Peterson provided advice on study design and data analysis. David A. Haidar, Rob D. Huang, and Patrick G. Minges contributed to data collection. David A. Haidar and William J. Peterson obtained institutional review board approval and exemption. David A. Haidar, Rob D. Huang, and William J. Peterson developed survey. Patrick G. Minges, Jennifer Carnell, Jason T. Nomura, John Bailitz, Jeremy S. Boyd, Megan M. Leo, E. Liang Liu, Youyou Duanmu, Josie Acuña, Ross Kessler, Marco F. Elegante, Mathew Nelson, Rachel B. Liu, Resa E. Lewiss, and Arun Nagdev all completed surveys and participated in discussion and revision of survey along with David A. Haidar, Rob D. Huang, and William J. Peterson. David A. Haidar and William J. Peterson analyzed the data. David A. Haidar, William J. Peterson, and Rob D. Huang drafted manuscript and prepared the tables and figures. All listed authors contributed substantially to the editing of the manuscript and conduct of this study. David A. Haidar takes responsibility of corresponding author.

## CONFLICT OF INTEREST

JC provides consulting services for Caption Health, Inc. JTN provides consulting services for Philips Healthcare and reports grant money from BMS to conduct research. JB reports grant money from Caption Health, Inc. to conduct research. RBL provides consulting services for Philips Healthcare, Caption Health, Inc., and Butterfly Network, Inc. REL provides consulting services for Echonous. The other authors declare no potential conflict of interest.

## Supporting information


Appendix S1
