# Supplementary material for: A consensus list of ultrasound competencies for graduating emergency medicine residents
Source: AEM Educ Train. 2022 Nov 21;6(6):e10817. doi: 10.1002/aet2.10817 (PMC9677397; doi:10.1002/aet2.10817)
Supplement: Supplementary file 1 — Appendix S1 [file AET2-6-e10817-s001.pdf]

Supplemental Table 1. Results of Round 1 and Round 2 of Delphi Voting

General Principles	Round 1 Mean (SD)	Round 1 Level of Agreement	Round 2 Mean (SD)	Round 2 Level of Agreement
Identifies need to accurately label images with patient information	4.53 (0.52)	High	4.75 (0.58)	High
Places patient in appropriate position	4.07 (0.59)	High	4.56 (0.51)	High
Explains the exam to be performed to the patient	4.27 (0.8)	High	4.56 (0.51)	High
Selects appropriate probe	4.80 (0.41)	High	5.00 (0.00)	High
Selects appropriate exam pre-set	4.20 (0.68)	High	4.69 (0.48)	High
Adjusts depth to adequately identify all relevant anatomy and pathology	4.67 (0.62)	High	4.88 (0.34)	High
Adjusts gain to appropriately identify all relevant structures and pathology	4.73 (0.46)	High	4.88 (0.34)	High
Assures proper cleaning of probe before and after use	4.67 (0.49)	High	4.94 (0.25)	High
Relays findings to patient care team	4.80 (0.41)	High	4.88 (0.34)	High
Appropriately documents all findings in the medical record	4.87 (0.35)	High	5.00 (0.00)	High

Physics	Round 1 Mean (SD)	Round 1 Level of Agreement	Round 2 Mean (SD)	Round 2 Level of Agreement
Identifies beam width artifact	3.40 (0.91)	Low	3.50 (0.73)	Low
Identifies posterior acoustic enhancement	4.40 (0.63)	High	4.75 (0.45)	High
Identifies acoustic shadowing	4.80 (0.41)	High	4.81 (0.54)	High
Identifies mirror artifact	3.93 (0.96)	Low	4.31 (0.48)	High
Identifies side lobe artifact	3.67 (0.9)	Low	3.75 (0.77)	Medium
Identifies aliasing	3.20 (1.01)	Low	3.44 (0.73)	Low

Trauma: Normal Anatomy	Round 1 Mean (SD)	Round 1 Level of Agreement	Round 2 Mean (SD)	Round 2 Level of Agreement
Identify liver	4.80 (0.77)	High	5.00 (0.00)	High
Identify kidney	5.00 (0.00)	High	5.00 (0.00)	High
Identify diaphragm	4.93 (0.26)	High	5.00 (0.00)	High
Identify spine	4.67 (0.62)	High	4.88 (0.50)	High
Identify Morison's pouch	5.00 (0.00)	High	5.00 (0.00)	High
Identify splenorenal space	5.00 (0.00)	High	5.00 (0.00)	High
Identify splenodiaphragmatic space	5.00 (0.00)	High	5.00 (0.00)	High
Identifies stomach	3.87 (0.92)	Low	3.81 (0.66)	Low
Identify paracolic gutter bilaterally	4.33 (0.82)	High	4.13 (0.81)	High
Identify pleural space bilaterally	4.87 (0.35)	High	5.00 (0.00)	High
Identify bladder in transverse and sagittal planes	4.93 (0.26)	High	4.88 (0.50)	High
Identify uterus in a female patient	4.87 (0.52)	High	4.81 (0.54)	High
Identify prostate in a male patient	4.13 (0.99)	Low	4.31 (0.70)	High
Identify Pouch of Douglas in a female patient	4.67 (0.72)	High	4.88 (0.34)	High
Identify recto-vesicular space in a male patient	4.67 (0.72)	High	4.75 (0.58)	High
Identify ribs	4.80 (0.56)	High	4.88 (0.34)	High
Identify lung pleura	4.93 (0.26)	High	5.00 (0.00)	High
Identify lung sliding	4.93 (0.26)	High	5.00 (0.00)	High

Trauma: Pathology	Round 1 Mean (SD)	Round 1 Level of Agreement	Round 2 Mean (SD)	Round 2 Level of Agreement
Intra-abdominal hemorrhage - Identify the anechoic appearance of intra-abdominal free fluid	5.00 (0.00)	High	5.00 (0.00)	High
Intra-abdominal hemorrhage - Identify the hypoechoic/mixed echogenic appearance of clotting intraperitoneal blood	4.27 (0.8)	High	4.44 (0.51)	High
Intra-abdominal hemorrhage - Identify potential spaces where intra-abdominal blood can accumulate	4.93 (0.26)	High	5.00 (0.00)	High
Intra-abdominal hemorrhage - Identify fascial tissue defects under ultrasound guidance after penetrating injury	2.53 (1.13)	Low	2.19 (0.83)	Low
Pneumothorax - Identify loss of lung sliding	5.00 (0.00)	High	5.00 (0.00)	High
Pneumothorax - Identify the appearance of the lung point	4.13 (0.92)	Low	4.38 (0.81)	High
Pneumothorax - Demonstrate the barcode sign in M-mode	4.00 (0.85)	Low	3.69 (0.70)	Medium
Pleural Effusion/Hemothorax - Identify the pleural space where pleural fluid will accumulate	4.87 (0.35)	High	4.88 (0.50)	High
Pleural Effusion/Hemothorax - Identify the appearance of anechoic pleural fluid	4.87 (0.35)	High	4.88 (0.50)	High
Pleural Effusion/Hemothorax - Identify the appearance of mixed-echogenicity complex pleural fluid	4.2 (0.86)	High	4.06 (0.68)	High
Pleural Effusion/Hemothorax - Identify the spine sign	4.53 (0.74)	High	4.81 (0.54)	High

Aorta: Normal Anatomy	Round 1 Mean (SD)	Round 1 Level of Agreement	Round 2 Mean (SD)	Round 2 Level of Agreement
Identify aorta in transverse, sagittal, and coronal plane	4.80 (0.41)	High	4.75 (0.58)	High
Identify spine	4.80 (0.41)	High	4.81 (0.54)	High
Identify inferior vena cava (IVC)	4.93 (0.26)	High	4.88 (0.50)	High
Identify celiac axis	4.00 (0.93)	Low	4.19 (0.83)	High
Identify superior mesenteric artery (SMA)	4.33 (0.82)	High	4.44 (0.73)	High
Identify aortic bifurcation	4.87 (0.35)	High	4.69 (0.60)	High
Identify renal arteries	2.93 (0.96)	Low	3 (0.89)	Low
Identify left renal vein	3.27 (1.03)	Low	3.31 (1.08)	Low
Identify splenic vein	3.4 (1.12)	Low	3.44 (1.03)	Low

Aorta: Pathology	Round 1 Mean (SD)	Round 1 Level of Agreement	Round 2 Mean (SD)	Round 2 Level of Agreement
Aortic Aneurysm - Measure abdominal aorta in transverse in proximal, mid, and distal abdomen	4.67 (0.62)	High	4.69 (0.48)	High
Aortic Aneurysm - Measure outer to outer wall in anterior to posterior plane	4.8 (0.56)	High	4.88 (0.34)	High
Aortic Aneurysm - Measure outer to outer wall	4.6 (0.51)	High	4.88 (0.34)	High
Aortic Aneurysm - Identifies aortic atheromatous plaques	3.13 (1.06)	Low	3.19 (0.83)	Low
Aortic Dissection - Identify aortic dissection flap	4.40 (0.83)	High	4.44 (0.63)	High
Aortic Dissection - Evaluate the area of dissection with color flow	3.13 (0.83)	Low	3.06 (0.85)	Low
Aortic Dissection - Identify variations in flow across the dissection flap	2.40 (0.99)	Low	2.63 (1.02)	Low

Aortic Dissection - Evaluate for level of dissection extension from aortic root to aortic bifurcation	3.13 (1.19)	Low	3.06 (0.85)	Low
Aortic Dissection - Measure aortic root	4.40 (0.83)	High	4.31 (0.70)	High
Aortic Dissection - Evaluate for level of dissection extension up carotid arteries bilaterally	2.87 (0.83)	Low	2.81 (0.40)	Low

Lung: Normal Anatomy	Round 1 Mean (SD)	Round 1 Level of Agreement	Round 2 Mean (SD)	Round 2 Level of Agreement
Normal Anatomy - Identify the different zones of the lung (Upper/Mid/Lower)	4.33 (0.82)	High	4.13 (0.81)	High
Normal Anatomy - Identify A-lines	4.8 (0.56)	High	4.69 (0.60)	High
Normal Anatomy - Identify Z-lines	3.53 (1.25)	Low	3.44 (0.96)	Low

Lung: Pathology	Round 1 Mean (SD)	Round 1 Level of Agreement	Round 2 Mean (SD)	Round 2 Level of Agreement
Interstitial Pulmonary Fluid - Identify B-lines	5.00 (0.00)	High	4.94 (0.25)	High
Interstitial Pulmonary Fluid - identify pulmonary edema patter of diffuse bilateral B-lines	4.87 (0.52)	High	n/a	n/a
Interstitial Pulmonary Fluid - Identify acute respiratory distress syndrome (ARDS) pattern of patchy bilateral B-lines	3.73 (0.80)	Low	n/a	n/a
Interstitial Pulmonary Fluid – Identify interstitial lung disease and interstitial pulmonary fluid pattern of diffuse B-lines at lung bases	3.0 (1.00)	Low	n/a	n/a
Identify differential for diffuse bilateral B-line pattern	n/a	n/a	4.81 (0.40)	High
Identify differential for focal bilateral B-line pattern	n/a	n/a	4.69 (0.48)	High
Pneumonia - Identify pneumonia pattern of focal B-line appearance	4.4 (0.51)	High	4.56 (0.51)	High

Pneumonia - Identify lung consolidation	4.53 (0.52)	High	4.63 (0.50)	High
Pneumonia - Identify subpleural effusion	4.13 (0.64)	High	4.06 (0.77)	High
Pneumonia - Identify static air bronchograms	3.33 (1.05)	Low	3.44 (0.81)	Low
Pneumonia - Identify mobile air bronchograms	3.4 (1.12)	Low	3.44 (0.81)	Low

Cardiac: Normal Anatomy	Round 1 Mean (SD)	Round 1 Level of Agreement	Round 2 Mean (SD)	Round 2 Level of Agreement
Identify right atrium (RA) in apical four chamber and subxiphoid views	4.87 (0.35)	High	4.88 (0.34)	High
Identify right ventricle (RV) in parasternal long, parasternal short, apical four chamber, and subxiphoid views	5.00 (0.00)	High	4.94 (0.25)	High
Identify left atrium (LA) in parasternal long, apical four chamber, and subxiphoid views	4.8 (0.56)	High	4.94 (0.25)	High
Identify left ventricle (LV) in parasternal long, parasternal short, apical four chamber, and subxiphoid views	5.00 (0.00)	High	4.94 (0.25)	High
Identify aortic outflow tract in parasternal long and apical four chamber views	4.8 (0.41)	High	4.88 (0.34)	High
Identify descending thoracic aorta in parasternal long view	4.6 (0.63)	High	4.75 (0.58)	High
Identify mitral valve in parasternal long, parasternal short, apical four chamber, and subxiphoid views	4.73 (0.46)	High	4.94 (0.25)	High
Identify tricuspid valve in apical four chamber and subxiphoid views	4.6 (0.63)	High	4.75 (0.45)	High
Identify aortic valve in parasternal long, apical four chamber, and subxiphoid views	4.47 (0.83)	High	4.81 (0.40)	High
Identify papillary muscles	4.6 (0.63)	High	4.75 (0.58)	High
Identify cardiac apex in parasternal short	3.93 (0.8)	High	3.75 (0.77)	Medium
Identify pericardium	4.93 (0.26)	High	4.94 (0.25)	High
Identify IVC in long and short axis	4.87 (0.35)	High	4.88 (0.34)	High
Identify hepatic vein confluence with IVC	4.27 (0.96)	High	4.44 (0.51)	High

Identify structures using transesophageal echo via mid-esophageal 4 chamber, mid esophageal long axis, transgastric short axis, Bicaval, and thoracic aorta views	2.07 (0.80)	Low	2 (0.63)	Low

Cardiac: Pathology	Round 1 Mean (SD)	Round 1 Level of Agreement	Round 2 Mean (SD)	Round 2 Level of Agreement
Cardiac Arrest - Identify sonographic appearance of cardiac standstill	4.80 (0.56)	High	4.94 (0.25)	High
Cardiac Arrest - Identify sonographic appearance of ventricular fibrillation	4.27 (1.1)	High	4.19 (0.83)	High
Cardiac Arrest - Identify sonographic appearance of agonal cardiac activity	4.40 (0.74)	High	4.44 (0.63)	High
Decreased Ejection Fraction - Accurately characterize ejection fraction into hyperdynamic/normal//mildly depressed/moderately depressed/severely depressed using subjective interpretation of cardiac contraction	4.80 (0.41)	High	4.81 (0.4)	High
Pathology - Decreased Ejection Fraction - Level of Importance - Quantitatively measure LV Ejection Fraction	n/a	n/a	3.06 (1.12)	Low
Decreased Ejection Fraction - Calculate ejection fraction using E-point septal separation	3.53 (0.99)	Low	3.31 (0.87)	Low
Decreased Ejection Fraction - Calculate ejection fraction using fractional shortening	2.27 (0.96)	Low	2.00 (0.63)	Low
Decreased Ejection Fraction - Calculate the ejection fraction using Simpson's method of discs	1.80 (0.77)	Low	1.63 (0.72)	Low
Cardiac Tamponade - Identifies where pericardial fluid accumulates	4.93 (0.26)	High	4.88 (0.34)	High
Cardiac Tamponade - Identifies right atrial collapse	4.33 (0.82)	High	4.5 (0.63)	High
Cardiac Tamponade - Identifies right ventricular collapse	4.8 (0.41)	High	4.94 (0.25)	High
Cardiac Tamponade - Identifies plethoric IVC	5.00 (0.00)	High	4.94 (0.25)	High
Cardiac Tamponade - Identifies mitral inflow variation	2.87 (1.06)	Low	2.81 (0.54)	Low
Valvular - Identifies mitral regurgitation using color doppler assessment	3.07 (0.8)	Low	2.88 (0.62)	Low

Valvular - Identifies tricuspid regurgitation using color doppler assessment	3.00 (0.85)	Low	2.75 (0.58)	Low
Valvular - Identifies aortic regurgitation using color doppler assessment	3.00 (1.07)	Low	2.88 (0.62)	Low
Valvular -Identifies mitral stenosis using mitral valve area assessment	1.67 (0.72)	Low	1.44 (0.63)	Low
Valvular -Identifies mitral stenosis using continuity equation assessment	1.4 (0.51)	Low	1.25 (0.45)	Low
Valvular - Identifies mitral stenosis using proximal isovelocity surface area assessment	1.47 (0.52)	Low	1.25 (0.45)	Low
Valvular - Identifies aortic stenosis using jet velocity assessment	1.67 (0.62)	Low	1.56 (0.73)	Low
Valvular - Identifies aortic stenosis using peak gradient assessment	1.53 (0.52)	Low	1.44 (0.63)	Low
Valvular - Identifies aortic stenosis using aortic valve area assessment	1.53 (0.52)	Low	1.38 (0.50)	Low
Valvular - Identifies vegetation on valve	4.13 (0.74)	High	3.94 (0.57)	High
Valvular - Measures aortic root diameter	4.6 (0.51)	High	4.44 (0.63)	High
Valvular - Evaluates for aortic root excursion	2.13 (1.30)	Low	1.94 (0.77)	Low
Volume Assessment - Demonstrate evaluation of IVC collapsibility 2 centimeters inferior from the confluence of the hepatic veins	4.33 (1.11)	High	4.5 (0.52)	High
Volume Assessment - Identifies plethoric IVC	4.87 (0.35)	High	4.94 (0.25)	High
Volume Assessment - Identifies collapsed IVC	4.87 (0.35)	High	4.94 (0.25)	High
Volume Assessment - Identify common carotid and measure VTi for volume responsiveness	1.80 (0.68)	Low	1.63 (0.72)	Low
Volume Assessment - Measure peak velocity variation of common carotid artery to estimate preload	1.67 (0.72)	Low	1.31 (0.48)	Low
Elevated Right Heart Pressure - Identifies the D sign	5.00 (0.00)	High	4.94 (0.25)	High
Elevated Right Heart Pressure - Identifies an increased RV:LV ratio	4.93 (0.26)	High	4.88 (0.34)	High
Elevated Right Heart Pressure - Measures RV in apical 4 chamber	3.53 (1.46)	Low	2.75 (0.77)	Low
Elevated Right Heart Pressure - Measures LV in apical 4 chamber	3.53 (1.46)	Low	2.75 (0.77)	Low

Elevated Right Heart Pressure - Demonstrates a TAPSE measurement at the free RV tricuspid annulus	3.67 (0.49)	Low	3.56 (0.73)	Low
Elevated Right Heart Pressure - Demonstrates a RVSP measurement using continuous wave doppler	1.87 (0.74)	Low	1.63 (0.62)	Low
Myocardial Infarction - Identifies the septal wall of the heart on parasternal short	4.47 (0.74)	High	4.19 (0.91)	High
Myocardial Infarction - Identifies the anterior wall of the heart on parasternal short	4.40 (0.74)	High	4.00 (0.82)	High
Myocardial Infarction - Identifies the lateral wall of the heart on parasternal short	4.40 (0.74)	High	4.00 (0.82)	High
Myocardial Infarction - Identifies the posterior wall of the heart on parasternal short	4.33 (0.72)	High	4.00 (0.82)	High
Myocardial Infarction - Identifies the inferior wall of the heart on parasternal short	4.33 (0.72)	High	4.00 (0.82)	High
Myocardial Infarction - Identifies abnormal regional wall motion	n/a	n/a	3.88 (0.89)	Medium
Myocardial Infarction - Identifies hypokinesis of a regional wall	3.40 (0.91)	Low	3.19 (0.91)	Low
Myocardial Infarction - Identifies akinesis of a regional wall	3.80 (0.68)	Low	3.69 (0.79)	Low
Diastolic Dysfunction - Demonstrates a measurement of the E and A wave using pulse wave doppler	2.6 (1.24)	Low	2.25 (0.93)	Low
Diastolic Dysfunction - Identifies normal E:A ratio	2.47 (1.19)	Low	2.06 (0.77)	Low
Diastolic Dysfunction - Identifies abnormal E:A ratio	2.47 (1.19)	Low	2.06 (0.77)	Low
Diastolic Dysfunction - Performs tissue doppler imaging of the mitral annulus	2.07 (0.96)	Low	1.81 (0.54)	Low
Diastolic Dysfunction - Identifies normal e':a' ratio	2.07 (0.96)	Low	1.81 (0.54)	Low
Diastolic Dysfunction - Identifies abnormal e':a' ratio	2.07 (0.96)	Low	1.81 (0.54)	Low
Diastolic Dysfunction - Calculates E:e'	2.07 (0.96)	Low	1.69 (0.6)	Low
Diastolic Dysfunction - Estimates RV:LV ratio	3.67 (1.45)	Low	3.63 (1.50)	Medium
Diastolic Dysfunction - Obtains and Calculates septal and mitral annular excursion velocity	1.80 (0.86)	Low	1.50 (0.63)	Low
Structural Abnormalities - Identifies contrast moving across septal wall indicating atrial or ventricular septal defect	2.07 (0.80)	Low	1.94 (0.57)	Low

Structural Abnormalities - Identifies color flow across septal wall indicating atrial or ventricular septal defect	2.4 (1.12)	Low	1.94 (0.57)	Low
Structural Abnormalities - Recognizes contrast in RV in bubble study	2.6 (1.35)	Low	2.38 (0.89)	Low

Obstetrics & Gynecology: Normal Anatomy	Round 1 Mean (SD)	Round 1 Level of Agreement	Round 2 Mean (SD)	Round 2 Level of Agreement
Identifies uterus in transverse axis using transabdominal approach	4.73 (0.59)	High	4.94 (0.25)	High
Identifies uterus in the sagittal axis using the transabdominal approach	4.73 (0.59)	High	4.94 (0.25)	High
Identifies uterus in the coronal axis using the transvaginal approach	4.47 (0.83)	High	4.44 (0.89)	High
Identifies uterus in the sagittal axis using the transvaginal approach	4.47 (0.83)	High	4.44 (0.89)	High
Identifies bladder	4.87 (0.35)	High	4.94 (0.25)	High
Identifies ovaries	n/a	n/a	4.19 (0.66)	High
Identifies right ovary	4.00 (1.00)	Low	4.00 (0.82)	High
Identifies left ovary	4.00 (1.00)	Low	4.00 (0.82)	High
Identifies Pouch of Douglas	4.73 (0.59)	High	4.88 (0.34)	High
Identifies endometrial stripe	4.67 (0.49)	High	4.88 (0.34)	High
Identifies cervix	4.33 (0.82)	High	4.44 (0.81)	High
First Trimester Intrauterine Pregnancy - Identifies gestational sac	4.8 (0.56)	High	4.94 (0.25)	High
First Trimester Intrauterine Pregnancy - Identifies yolk sac	4.93 (0.26)	High	4.94 (0.25)	High
First Trimester Intrauterine Pregnancy - Identifies fetal pole	4.8 (0.41)	High	4.88 (0.34)	High
First Trimester Intrauterine Pregnancy - Identifies fetal heart rate	4.6 (0.83)	High	4.75 (0.77)	High
First Trimester Intrauterine Pregnancy - Measure fetal heart rate using M-mode	4.53 (0.83)	High	4.69 (0.79)	High

First Trimester Intrauterine Pregnancy - Identifies thickness of myometrium between gestation sac and the uterine wall	3.73 (1.22)	Low	3.69 (1.01)	Low
First Trimester Intrauterine Pregnancy - Performs crown-rump length measurement to estimate gestational age	3.8 (1.37)	Low	3.81 (0.83)	High
First Trimester Intrauterine Pregnancy - identifies molar pregnancy	3.67 (1.11)	Low	3.5 (0.73)	Low
Third Trimester Intrauterine Pregnancy - Demonstrates use of pulse wave doppler to evaluate umbilical artery blood flow in high-risk pregnancies	1.47 (0.52)	Low	1.19 (0.4)	Low
Third Trimester Intrauterine Pregnancy - Calculates Estimated Fetal Weights	1.47 (0.52)	Low	1.13 (0.34)	Low
Third Trimester Intrauterine Pregnancy - Determines fetal lie	3.07 (1.49)	Low	3.13 (1.15)	Low

Obstetrics & Gynecology: Pathology	Round 1 Mean (SD)	Round 1 Level of Agreement	Round 2 Mean (SD)	Round 2 Level of Agreement
Ovarian Cyst - Identifies cystic structure within ovaries	3.67 (0.72)	Low	3.63 (0.72)	Low
Ovarian Cyst - Identifies simple cyst (anechoic vs hypoechoic)	3.4 (0.83)	Low	3.44 (0.73)	Low
Ovarian Cyst - Identifies complex cyst (loculated, anechoic, hypoechoic or hyperechoic)	3.27 (0.80)	Low	3.38 (0.81)	Low
Ovarian Cyst - identifies adnexal mass	3.53 (0.74)	Low	2.44 (0.81)	Low
Ovarian Cyst - Identified tubo-ovarian abscess	3.07 (0.8)	Low	3.19 (0.54)	Low
Ovarian Cyst - Identifies echogenic fluid in the cul-de-sac	4.53 (0.64)	High	4.75 (0.45)	High
Ovarian Torsion - Demonstrates use of power doppler to evaluate ovarian blood flow	2.8 (0.68)	Low	2.44 (0.81)	Low
Ovarian Torsion - Demonstrates use of pulse wave doppler to evaluate ovarian blood flow	2.67 (0.98)	Low	2.31 (0.79)	Low
Ovarian Torsion - Identifies arterial wave form on pulse wave doppler of ovarian vasculature	2.53 (0.92)	Low	2.25 (0.68)	Low

Ovarian Torsion - Identifies venous wave form on pulse wave doppler of ovarian vasculature	2.4 (0.63)	Low	2.19 (0.66)	Low
Ectopic Pregnancy - Identifies empty uterus in setting of positive pregnancy test	5.00 (0.00)	High	4.94 (0.25)	High
Ectopic Pregnancy - Identifies pseudogestional sac in setting of positive pregnancy test	4.27 (1.16)	High	4.44 (0.51)	High
Ectopic Pregnancy - Identifies yolk sac/fetal pole in a non-uterine location	4.47 (0.92)	High	4.44 (0.63)	High
Ectopic Pregnancy - Identifies free fluid in the Pouch of Douglas	5.00 (0.00)	High	4.94 (0.25)	High
Ectopic Pregnancy - Identifies free fluid in Morison's pouch	5.00 (0.00)	High	4.88 (0.34)	High

Testicular: Normal Anatomy	Round 1 Mean (SD)	Round 1 Level of Agreement	Round 2 Mean (SD)	Round 2 Level of Agreement
Normal Anatomy - Identifies testicle	4.40 (0.74)	High	4.38 (0.89)	High
Normal Anatomy - Identifies epididymis	3.73 (0.96)	Low	3.56 (1.15)	Low
Normal Anatomy - Identifies scrotal raphae	2.67 (1.11)	Low	2.38 (0.81)	Low
Normal Anatomy - Identifies mediastinum testis	2.73 (1.1)	Low	2.38 (0.89)	Low

Testicular: Pathology	Round 1 Mean (SD)	Round 1 Level of Agreement	Round 2 Mean (SD)	Round 2 Level of Agreement
Testicular Torsion - Demonstrates use of power doppler to evaluate testicular blood flow	3.67 (1.05)	Low	3.5 (0.73)	Low
Testicular Torsion - Demonstrates use of pulse-wave doppler to evaluate testicular blood flow	2.93 (1.1)	Low	2.69 (1.01)	Low
Testicular Torsion - Identifies arterial wave form on pulse wave doppler of testicular vasculature	2.80 (1.15)	Low	2.56 (0.81)	Low

Testicular Torsion - Identifies venous wave form on pulse wave doppler of testicular vasculature	2.67 (0.98)	Low	2.50 (0.73)	Low
Testicular Torsion - Demonstrates side-by-side view of testes to compare blow flow	3.73 (1.03)	Low	3.38 (1.09)	Low
Hydrocele/Varicocele/Hernia - Identifies hydrocele	3.47 (1.25)	Low	3.06 (0.93)	Low
Hydrocele/Varicocele/Hernia - Identifies varicocele	2.93 (1.16)	Low	2.50 (0.89)	Low
Hydrocele/Varicocele/Hernia - Identifies contents of inguinal hernia present in scrotum	3.20 (1.08)	Low	2.81 (1.05)	Low
Epididymitis/Orchitis - Identifies hypervascular power doppler pattern of epididymitis	3.20 (1.08)	Low	2.69 (1.08)	Low
Epididymitis/Orchitis - Identifies heterogenous echotexture of epididymitis	2.87 (1.3)	Low	2.56 (0.89)	Low
Epididymitis/Orchitis - Identifies peri-epididymal fluid	2.73 (1.28)	Low	2.31 (0.7)	Low
Epididymitis/Orchitis - Identifies hypervascular power doppler pattern of orchitis	3.00 (1.07)	Low	2.63 (0.96)	Low
Epididymitis/Orchitis - Identifies heterogenous echotexture of orchitis	2.73 (1.22)	Low	2.31 (0.70)	Low

Ocular: Normal Anatomy	Round 1 Mean (SD)	Round 1 Level of Agreement	Round 2 Mean (SD)	Round 2 Level of Agreement
Identifies anterior chamber	4.67 (0.62)	High	4.75 (0.45)	High
Identifies posterior chamber	4.87 (0.35)	High	4.56 (1.03)	High
Identifies pupil	4.07 (1.03)	Low	4.19 (0.66)	High
Identifies lens	4.6 (0.91)	High	4.56 (0.63)	High
Identifies optic nerve sheath	4.53 (0.83)	High	4.63 (0.50)	High
Identifies macula	3.4 (1.18)	Low	3.25 (1.13)	Low

Ocular: Pathology	Round 1 Mean (SD)	Round 1 Level of Agreement	Round 2 Mean (SD)	Round 2 Level of Agreement
Posterior Chamber - Identifies vitreous hemorrhage	4.40 (0.63)	High	4.44 (0.63)	High
Posterior Chamber - Identifies vitreous detachment	4.53 (0.64)	High	4.63 (0.5)	High
Posterior Chamber - Identifies retinal detachment	4.8 (0.41)	High	4.81 (0.4)	High
Posterior Chamber - Distinguishes vitreous from retinal detachment via visualization of optic nerve sheath	4.53 (0.64)	High	4.25 (0.77)	High
Posterior Chamber - Demonstrates dynamic evaluation of the eye via patient eye movement	4.33 (0.82)	High	4.50 (0.52)	High
Posterior Chamber - Differentiates macula on and macula off retinal detachment	2.93 (1.16)	Low	2.75 (1.13)	Low
Other - Measures optic nerve sheath accurately (3 mm from posterior chamber)	3.73 (0.96)	Low	3.50 (0.97)	Low
Other – Identifies globe rupture	4.07 (0.88)	High	3.88 (0.89)	High

Neurology	Round 1 Mean (SD)	Round 1 Level of Agreement	Round 2 Mean (SD)	Round 2 Level of Agreement
Lumbar Puncture - Uses transcranial doppler to evaluate cerebral blood flow velocities	1.53 (0.64)	Low	1.19 (0.4)	Low
Lumbar Puncture - Identify spinous process	3.33 (1.23)	Low	2.75 (1.06)	Low
Lumbar Puncture - Identify ligamentum flavum	2.33 (0.82)	Low	2.00 (0.73)	Low
Lumbar Puncture - Identify laminae	2.40 (0.74)	Low	2.00 (1.03)	Low
Lumbar Puncture - Identify epidural space	2.20 (0.77)	Low	1.75 (0.68)	Low
Lumbar Puncture - Identify subarachnoid space	2.07 (0.59)	Low	1.75 (0.68)	Low
Lumbar Puncture - Identify posterior longitudinal ligament	2.07 (0.59)	Low	2.00 (0.73)	Low

Venous: Normal Anatomy	Round 1 Mean (SD)	Round 1 Level of Agreement	Round 2 Mean (SD)	Round 2 Level of Agreement
Identifies greater saphenous vein	4.53 (0.83)	High	4.69 (0.6)	High
Identifies common femoral vein	4.8 (0.41)	High	4.81 (0.54)	High
Identifies deep femoral vein	4.73 (0.46)	High	4.75 (0.58)	High
Identifies superficial femoral vein	4.73 (0.46)	High	4.75 (0.58)	High
Identifies popliteal vein	4.73 (0.46)	High	4.81 (0.54)	High
Identifies popliteal trifurcation (anterior tibial, posterior tibial, peroneal)	4.2 (0.56)	High	4.13 (0.72)	High

Venous: Pathology	Round 1 Mean (SD)	Round 1 Level of Agreement	Round 2 Mean (SD)	Round 2 Level of Agreement
DVT - Identifies compressibility of a normal vessel	4.87 (0.35)	High	4.81 (0.54)	High
DVT - Identifies lack of compressibility in the setting of a DVT	4.87 (0.35)	High	4.88 (0.50)	High
DVT - Performs compression at areas of venous bifurcation	4.33 (0.98)	High	4.25 (0.77)	High
DVT - Utilizes color augmentation for evaluation of venous structures	3.13 (1.13)	Low	2.94 (0.93)	Low
DVT - Identifies presence of DVT in upper extremity	n/a	n/a	3.00 (1.03)	Low
DVT - Identifies presence of DVT in lower extremity	n/a	n/a	4.69 (0.60)	High

Biliary: Normal Anatomy	Round 1 Mean (SD)	Round 1 Level of Agreement	Round 2 Mean (SD)	Round 2 Level of Agreement
Identifies liver	4.87 (0.35)	High	4.94 (0.25)	High
Identifies gallbladder in long axis	4.87 (0.35)	High	4.94 (0.25)	High

Identifies gallbladder in short axis	4.87 (0.35)	High	4.94 (0.25)	High
Identifies main lobar fissure	3.47 (0.99)	Low	3.63 (0.96)	Low
Identifies portal triad	4.67 (0.49)	High	4.63 (0.50)	High
Identifies portal vein	4.67 (0.49)	High	4.56 (0.51)	High
Identifies hepatic artery	4.27 (0.88)	High	4.06 (0.57)	High
Identifies common bile duct	4.33 (0.62)	High	4.19 (0.75)	High

Biliary: Pathology	Round 1 Mean (SD)	Round 1 Level of Agreement	Round 2 Mean (SD)	Round 2 Level of Agreement
Gallstones - Identifies gallstones	4.93 (0.26)	High	4.94 (0.25)	High
Gallstones - Identifies gallstone characteristics of echogenicity, shadowing, and mobility	4.87 (0.35)	High	4.94 (0.25)	High
Gallstones - Identifies Wall-Echo-Shadow (WES) sign	4.27 (0.8)	High	4.13 (0.62)	High
Gallstones - Identifies gallbladder sludge	4.13 (0.83)	Medium	4 (0.73)	High
Cholecystitis - Identifies pericholecystic fluid	4.8 (0.56)	High	4.81 (0.54)	High
Cholecystitis - Identifies increase size of gallbladder wall	4.67 (0.62)	High	4.63 (0.72)	High
Cholecystitis - Measures anterior gallbladder wall in short axis	4.4 (0.83)	High	4.44 (0.73)	High
Cholecystitis - Identifies sonographic murphy's sign	4.33 (0.98)	High	4.44 (0.73)	High
Choledocholithiasis/cholangitis - Identifies enlarged common bile duct	4.2 (0.68)	High	4.13 (0.96)	High
Choledocholithiasis/cholangitis - Measures common bile duct	3.93 (0.70)	High	4.06 (0.77)	High
Choledocholithiasis/cholangitis - Identifies stone in common bile duct	2.73 (0.96)	Low	2.56 (0.81)	Low

Renal: Normal Anatomy	Round 1 Mean (SD)	Round 1 Level of Agreement	Round 2 Mean (SD)	Round 2 Level of Agreement
Identifies renal cortex	4.53 (0.52)	High	4.69 (0.48)	High
Identifies renal medulla	4.4 (0.63)	High	4.69 (0.48)	High
Identifies renal pelvis	4.87 (0.35)	High	4.88 (0.34)	High
Identifies ureter	4.33 (0.82)	High	4.13 (0.5)	High
Identifies bladder	4.87 (0.35)	High	4.88 (0.34)	High
Identifies ureteral papillae on posterior surface of bladder	2.93 (1.39)	Low	2.69 (1.25)	Low

Renal: Pathology	Round 1 Mean (SD)	Round 1 Level of Agreement	Round 2 Mean (SD)	Round 2 Level of Agreement
Hydronephrosis - Identifies mild hydronephrosis	4.73 (0.46)	High	4.75 (0.45)	High
Hydronephrosis - Identifies moderate hydronephrosis	4.93 (0.26)	High	4.94 (0.25)	High
Hydronephrosis - Identifies severe hydronephrosis	4.93 (0.26)	High	4.94 (0.25)	High
Hydronephrosis -Identifies mimics of hydronephrosis	n/a	n/a	4.25 (0.86)	High
Hydronephrosis - Uses color to differentiate hydronephrosis from vasculature	n/a	n/a	4.56 (0.51)	High
Kidney Stone - Identifies intra-renal stones	2.80 (0.68)	Low	2.75 (0.58)	Low
Kidney Stone - Identifies stone characteristics of echogenicity and shadowing	3.53 (1.19)	Low	3.63 (0.89)	Low
Kidney Stone - Identifies renal stone at ureterovesicular junction	3.67 (1.18)	Low	3.56 (0.81)	Low
Kidney Stone - Identifies twinkle artifact when performing color doppler at ureterovesicular junction	3.13 (1.13)	Low	3.19 (0.83)	Low
Other - Demonstrates renal restrictive indices using pulse wave doppler	1.8 (0.56)	Low	1.44 (0.51)	Low
Other - Identifies renal cysts	3.8 (0.86)	Low	3.88 (1.02)	Medium

Other - Differentiates simple vs complex cyst	3.07 (1.03)	Low	3.19 (1.17)	Low
Other - Identify mixed echogenicity of renal pelvices in acute pyelonephritis	2.33 (0.62)	Low	1.81 (0.66)	Low
Other - identify debris in renal collecting system indicative of acute pyelonephritis	2.2 (0.56)	Low	1.88 (0.81)	Low
Other - Identify fluid collection concerning for development of acute lobar nephronia	1.87 (0.74)	Low	1.44 (0.63)	Low

Musculoskeletal: Normal Anatomy	Round 1 Mean (SD)	Round 1 Level of Agreement	Round 2 Mean (SD)	Round 2 Level of Agreement
Identifies muscle appearance	4.53 (0.92)	High	4.5 (0.82)	High
Identifies tendon appearance - fibrillar, attaches to muscle/bone	4.47 (1.06)	High	4.56 (0.81)	High
Identifies peripheral nerve appearance - anisotropy	4.13 (0.92)	High	4.19 (0.66)	High
Identifies peripheral nerve appearance - hyperechoic honeycomb	4.2 (0.86)	High	4.31 (0.60)	High
Identifies normal appearance of dermis	4 (1.13)	Low	4.13 (0.89)	High
Identifies appearance of lymph node	4.2 (0.86)	High	4.31 (0.60)	High
Identifies bone as hyperechoic line in longitudinal and transverse planes	4.53 (0.92)	High	4.69 (0.79)	High

Musculoskeletal: Pathology	Round 1 Mean (SD)	Round 1 Level of Agreement	Round 2 Mean (SD)	Round 2 Level of Agreement
General - Identifies breaks in cortex to indicate fracture	3.87 (1.13)	Low	3.56 (0.73)	Low
General - Identifies joint dislocation	4.13 (0.74)	High	3.88 (0.62)	High
General - Identifies successful joint reduction	4.07 (0.8)	Medium	3.75 (0.58)	Medium
General - Identifies joint effusion	4.67 (0.49)	High	4.81 (0.40)	High
Soft Tissue - Identifies cobblestoning	4.8 (0.41)	High	4.88 (0.34)	High

Soft Tissue - Identifies other causes of cobblestoning - lymphedema/edema	4.73 (0.46)	High	4.81 (0.54)	High
Soft Tissue - Identifies appearance of abscess	4.87 (0.35)	High	4.94 (0.25)	High
Soft Tissue - Uses color to help identify abscess	n/a	n/a	3.88 (1.15)	Low
Soft Tissue - Identifies air echoes as sign of necrotizing fasciitis	4.6 (0.51)	High	4.5 (0.82)	High
Soft Tissue - Identifies foreign objects in subcutaneous tissue	4.27 (0.59)	High	3.94 (0.77)	High
Shoulder - Identifies humeral head	4.40 (0.91)	High	3.88 (0.96)	High
Shoulder - Identifies glenoid	4.33 (0.90)	High	3.88 (0.96)	High
Shoulder - Identifies anterior shoulder dislocation	4.2 (0.86)	High	3.75 (1.00)	Medium
Shoulder - Identifies posterior shoulder dislocation	4.13 (0.83)	High	3.69 (1.01)	Low
Shoulder - Identifies rotator cuff tear	1.93 (0.8)	Low	1.63 (0.62)	Low
Shoulder - Confirms successful reduction on post-reduction ultrasound	3.87 (0.92)	High	3.44 (0.96)	Low
Knee - Identifies patella	4.27 (0.8)	High	3.94 (1.00)	High
Knee - Identifies femur	4.27 (0.8)	High	3.94 (1.00)	High
Knee - Identifies tibia	4.27 (0.8)	High	3.94 (1.00)	High
Knee - Identifies patellar tendon	4.27 (0.8)	High	3.94 (1.00)	High
Knee - Identifies medial/lateral meniscus	2.33 (0.82)	Low	1.75 (0.58)	Low
Knee - Identifies patellar tendon rupture	3.93 (0.7)	Medium	3.38 (0.96)	Low
Ankle - Identifies medial/lateral malleolus	3 (0.93)	Low	3.19 (1.05)	Low
Ankle - Identifies calcaneus	3.13 (0.92)	Low	3.19 (1.05)	Low
Ankle - Identifies tarsal bones	2.6 (0.74)	Low	2.31 (0.87)	Low
Ankle - Identifies achilles tendon	4.07 (0.70)	High	4.06 (0.68)	High

Ankle - Identifies achilles tendon rupture	4.13 (0.74)	High	4.06 (0.57)	High

Head & Neck	Round 1 Mean (SD)	Round 1 Level of Agreement	Round 2 Mean (SD)	Round 2 Level of Agreement
Identifies appearance of peritonsillar abscess using endocavitary probe	3.93 (0.59)	High	3.75 (0.58)	High
Identifies presence of intra-oral abscesses	n/a	n/a	3.56 (0.51)	Low

Bowel: Normal Anatomy	Round 1 Mean (SD)	Round 1 Level of Agreement	Round 2 Mean (SD)	Round 2 Level of Agreement
Identifies small bowel	3.47 (0.92)	Low	3.56 (0.73)	Low
Identifies plica circularis	2.87 (0.92)	Low	3.06 (0.93)	Low
Identifies colon - air interference	3.2 (0.68)	Low	3.31 (0.7)	Low
Identifies haustra	2.93 (0.70)	Low	3 (0.89)	Low
Identifies blind-ending appendix	3.27 (0.70)	Low	3.06 (0.77)	Low
Identifies iliac artery	3.4 (0.91)	Low	3.25 (0.68)	Low
Identifies psoas muscle	3.13 (0.74)	Low	2.94 (0.57)	Low

Bowel: Pathology	Round 1 Mean (SD)	Round 1 Level of Agreement	Round 2 Mean (SD)	Round 2 Level of Agreement
Small Bowel Obstruction - Measures small bowel wall	3.27 (0.96)	Low	3.00 (0.73)	Low
Small Bowel Obstruction - Measures small bowel diameter	3.73 (1.03)	Low	3.31 (0.79)	Low
Small Bowel Obstruction - Identifies to and fro peristalsis as sign of small bowel obstruction	3.93 (1.1)	Low	3.94 (0.77)	High
Appendicitis - Evaluates appendix with compression	3.47 (0.74)	Low	3.19 (0.83)	Low

Appendicitis - Identifies appendicolith	3.27 (0.96)	Low	3 (0.63)	Low
Appendicitis - Identifies free fluid surround appendix	3.53 (0.99)	Low	3.38 (0.89)	Low
Intussusception - Identifies hypoechoic edema around bowel wall	3.07 (0.70)	Low	2.81 (0.66)	Low
Intussusception - Identifies layers of hypoechoic and hyperechoic bowel wall indicating telescoping	3.00 (0.76)	Low	2.88 (0.81)	Low
Other - Measures pylorus length and width	2.67 (0.98)	Low	2.38 (0.62)	Low
Other - Identifies hypertrophic pyloric stenosis	2.73 (1.03)	Low	2.25 (0.58)	Low
Other - Identifies ingested foreign bodies in stomach	2.2 (0.86)	Low	2.06 (0.68)	Low

Airway	Round 1 Mean (SD)	Round 1 Level of Agreement	Round 2 Mean (SD)	Round 2 Level of Agreement
General Principles - Identify thyroid cartilage in transverse and longitudinal plane	3.2 (1.21)	Low	3.06 (1.00)	Low
General Principles - Identify cricoid cartilage in transverse and longitudinal plane	3.33 (1.23)	Low	3.06 (1.00)	Low
General Principles - Identify trachea and esophagus in transverse plane	3.4 (1.3)	Low	3.31 (0.95)	Low
General Principles - Identify thyroid	3.47 (1.25)	Low	3.38 (1.09)	Low
General Principles - Identify endotracheal tube in trachea post intubation	2.87 (1.30)	Low	2.69 (0.87)	Low
General Principles - Identifies cricothyroid membrane for cricothyroidotomy	3.47 (1.13)	Low	3.25 (0.93)	Low
General Principles – Identifies endotracheal tube (ETT) incorrectly placed in the esophagus	3.27 (1.16)	Low	2.88 (0.96)	Low
General Principles - Confirm airway placement by evaluating diaphragm motion	2.53 (1.19)	Low	2.19 (0.83)	Low
General Principles - Utilizes color doppler imaging to identify placement of ETT	1.80 (0.86)	Low	1.56 (0.63)	Low

Procedures	Round 1 Mean (SD)	Round 1 Level of Agreement	Round 2 Mean (SD)	Round 2 Level of Agreement
Needle Guidance - Identifies and tracks needle in long axis	4.87 (0.35)	High	4.88 (0.50)	High
Needle Guidance - Identifies and tracks needle tip in short axis	4.93 (0.26)	High	4.75 (0.58)	High
Needle Guidance - Identifies important nearby structures	4.93 (0.26)	High	4.88 (0.5)	High
Needle Guidance - Confirms location of guidewire within vessel	4.73 (0.46)	High	4.88 (0.5)	High
Needle Guidance - Confirms location of catheter within vessel	4.60 (0.51)	High	4.81 (0.54)	High
Needle Guidance - Correctly identifies appropriate vessel	4.93 (0.26)	High	4.88 (0.50)	High
Needle Guidance - Correctly identifies size and location of vessel	4.87 (0.35)	High	4.88 (0.50)	High
Needle Guidance - Identifies back-walling of needle or guidewire in vessel	4.73 (0.59)	High	4.69 (0.60)	High
Needle Guidance - Identifies needle going through and through vessel	4.8 (0.41)	High	4.81 (0.54)	High
Fluid Drainage - Identifies anechoic or hypoechoic fluid pocket	4.93 (0.26)	High	4.88 (0.50)	High
Procedures - Can place internal jugular CVC under ultrasound guidance	5.00 (0.00)	High	4.94 (0.25)	High
Procedures - Can place subclavian CVC under ultrasound guidance	3.47 (1.13)	Low	3.38 (0.89)	Low
Procedures - Can place femoral vein CVC under ultrasound guidance	4.80 (0.56)	High	4.88 (0.34)	High
Procedures - Can place radial arterial line under ultrasound guidance	4.60 (0.51)	High	4.69 (0.48)	High
Procedures - Can cannulate vessel in short and long axis	4.67 (0.62)	High	4.75 (0.45)	High
Procedures - Can place femoral arterial line under ultrasound guidance	4.60 (0.63)	High	4.75 (0.45)	High
Procedures - Can drain peritonsillar abscess under ultrasound guidance	3.53 (0.99)	Low	3.00 (0.82)	Low
Procedures - Can drain peritonsillar abscess with ultrasound assistance	n/a	n/a	3.81 (0.75)	Low

Procedures - Can perform thoracentesis under ultrasound guidance	4.6 (0.74)	High	4.63 (0.50)	High
Procedures - Can perform paracentesis under ultrasound guidance	4.87 (0.35)	High	4.50 (0.63)	High
Procedures - Can perform paracentesis with ultrasound assistance	n/a	n/a	4.75 (0.45)	High
Procedures - Can perform arthrocentesis under ultrasound guidance	4.60 (0.51)	High	4.44 (0.73)	High
Procedures - Can perform lumbar puncture under ultrasound guidance	3.20 (1.08)	Low	2.81 (0.66)	Low
Procedures - Can perform pericardiocentesis under ultrasound guidance	4.60 (0.74)	High	4.75 (0.45)	High
Procedures - Can confirm placement of ETT after intubation under ultrasound guidance	2.47 (1.06)	Low	2.56 (1.03)	Low
Procedures - Can confirm joint dislocation under ultrasound guidance	3.60 (1.12)	Low	3.63 (0.81)	Medium
Procedures - Can perform nerve blocks under ultrasound guidance	4.07 (0.80)	Medium	4.19 (0.66)	High
Procedures - Can place peripheral venous line under ultrasound guidance	4.93 (0.26)	High	4.94 (0.25)	High
Procedures - Confirm REBOA placement using contrast enhanced US	1.4 (0.51)	Low	1.44 (0.51)	Low
Procedures - Identifies lung re-expansion and determine chest tube removal appropriateness	2.67 (1.23)	Low	2.44 (1.09)	Low
Procedures - Can perform intra-articular hip steroid injection under ultrasound guidance	2.13 (1.13)	Low	1.94 (0.85)	Low
Procedures - Identifies Nasogastric tube in stomach under ultrasound guidance	2.20 (0.68)	Low	2.06 (0.68)	Low
Procedures - Identifies intercostal space for chest tube placement	3.20 (1.15)	Low	3.00 (0.97)	Low
